# Tissue-specific Differentiation Potency of Mesenchymal Stromal Cells from Perinatal Tissues

**DOI:** 10.1038/srep23544

**Published:** 2016-04-05

**Authors:** Ahlm Kwon, Yonggoo Kim, Myungshin Kim, Jiyeon Kim, Hayoung Choi, Dong Wook Jekarl, Seungok Lee, Jung Min Kim, Jong-Chul Shin, In Yang Park

**Affiliations:** 1 Catholic Genetic Laboratory Center, Seoul St. Mary’s Hospital, College of Medicine, The Catholic University of Korea, Seoul, Republic of Korea; 2 Department of Laboratory Medicine, College of Medicine, The Catholic University of Korea, Seoul, Republic of Korea; 3 NAR Center, Inc., Daejeon Oriental Hospital of Daejeon University, Daejeon, Republic of Korea; 4 Department of Obstetrics and Gynecology, College of Medicine, The Catholic University of Korea, Seoul, Republic of Korea

## Abstract

Human perinatal tissue is an abundant source of mesenchymal stromal cells(MSCs) and lacks the ethical concerns. Perinatal MSCs can be obtained from various tissues as like amnion, chorion, and umbilical cord. Still, little is known of the distinct nature of each MSC type. In this study, we successfully isolated and cultured MSCs from amnion(AMSCs), chorion(CMSCs), and umbilical cord(UC-MSCs). Proliferation potential was different among them, that AMSCs revealed the lowest proliferation rate due to increased Annexin V and senescence-associated β-galactosidase positive cells. We demonstrated distinct characteristic gene expression according to the source of the original tissue using microarray. In particular, genes associated with apoptosis and senescence including *CDKN2A* were up-regulated in AMSCs. In CMSCs, genes associated with heart morphogenesis and blood circulation including *HTR2B* were up-regulated. Genes associated with neurological system processes including *NPY* were up-regulated in UC-MSCs. Quantitative RT-PCR confirmed the gene expression data. And *in vitro* differentiation of MSCs demonstrated that CMSCs and UC-MSCs had a more pronounced ability to differentiate into cardiomyocyte and neural cells, respectively. This study firstly demonstrated the innate tissue-specific differentiation potency of perinatal MSCs which can be helpful in choosing more adequate cell sources for better outcome in a specific disease.

Mesenchymal stromal cells (MSCs) have the potential for self-renewal, immunomodulation, and differentiation into mesoderm lineages *in vitro* and *in vivo*. Currently, MSCs are considered as an important resource in regard to regenerative medicine applications. They can be isolated from various tissue types, including bone marrow (BM), lung, fat, liver, cord blood, amniotic fluid, placenta, and umbilical cord. MSCs can also be obtained from human term perinatal tissues including the amnion and chorion^1^,^2^. MSCs obtained from the umbilical cord have revealed *in vitro* proliferative potential and immunoregulatory features^3^,^4^. Human term perinatal tissue is typically discarded after birth. So, these tissues can be effectively utilized for research and clinical applications without ethical violations. The tissue’s large size offers a high quantity of MSCs, while its appreciable immunomodulatory properties have positioned the tissue as the most promising resource for regenerative medicine^4^,^5^.

Human amnion-derived MSCs (AMSCs), chorion-derived MSCs (CMSC), and umbilical cord-derived MSCs (UC-MSCs) typically adhere to plastic; form fibroblast colony-forming units; elaborate specific surface antigen patterns CD90^+^, CD73^+^, CD105^+^,CD45^–^, CD34^–^, CD14^–^, and HLA-DR^–^; can differentiate one or more of the adipogenic, chondrogenic, osteogenic, or vascular/endothelial lineages; and represent an intermediate stage between adult and embryonic stem cells (ESCs)^5^,^6^,^7^. Differences between MSCs from various tissues have been reported, such as those between the soluble factor secretions and angiogenic/immuno-suppressive functions of AMSCs and CMSCs^8^. Chorionic-plate-derived MSCs and UC-MSCs differ in terms of their potential to differentiate into different cell types like adipocytes, osteocytes, and hepatocytes^9^. The origin or source of MSCs may determine their fate and functional characteristics. A better understanding of the distinct characteristics of various MSCs is needed before they can be used clinically.

As AMSCs, CMSC, and UC-MSCs can be simultaneously obtained from one donor, their inherent characteristics can be explored while minimizing interpersonal variations. However, little information is currently available regarding their different characteristics and gene expression profiles. Analyzing gene expression profiles is a powerful approach, and can be used to understand the propensity and capacity of MSCs from a particular source to differentiate towards a specific lineage. We postulated that MSCs from different sources would exhibit their own gene expression profiles that explain their distinctive characteristics, such as differentiation lineage and/or adoption of a certain cell fate.

This study had four purposes: 1) to isolate and characterize AMSCs, CMSCs, and UC-MSCs from different human term perinatal tissues; 2) to compare differences in cellular proliferation, immunophenotype, and mesodermal differentiation potential; 3) to examine their gene expression profiles and investigate their differences; and 4) to determine the differentiation potential of each MSC-type. Discovering the specific potency of the MSC types could lead to more efficient methods for selecting cells directed towards specific purposes.

## Results

### Isolation and proliferation of MSCs

**Figure 1:**
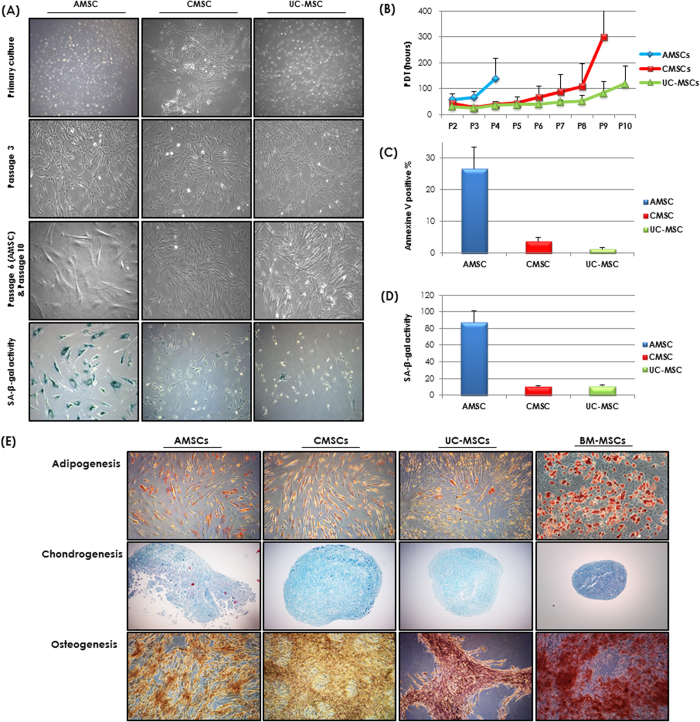
(**A**) Morphology of amnionic mesenchymal stromal cells(AMSCs) at primary culture, passage 3 and 6 (left), chorionic MSCs (CMSCs) (middle), and umbilical cord MSCs (UC-MSCs) (right) in primary culture, passage 3 and 10 (x40). (**B**) Comparison of population doubling time among AMSCs, CMSCs and UC-MSCs. (**C**) Evaluation of apoptosis by Annexin V-FITC/PI staining, followed by flow cytometry analysis. (**D**) Evaluation of Senescence by β-galactosidase activity assay. (**E**) Mesodermal differentiation potential of mesenchymal stromal cells derived from amnion (AMSCs), chorion (CMSCs) and umbilical cord (UC-MSCs). Adipogenesis (Oil red O), chondrogenesis (Alcian blue), osteogenesis (silver nitrate) (x100).

We successfully isolated and cultured MSCs from the amnion, chorion, and umbilical cord. At primary culture (P0), AMSCs were mixed with spindle-shaped cells and epithelial-like cells. Epithelial-like cells disappeared at P1 and spindle-shaped cells were maintained up to P2 (Fig. 1A). CMSCs and UC-MSCs presented spindle-shaped cells at P1 and maintained their morphology up to P9 or P10.Two AMSCs discontinued proliferation at P5 and one AMSC discontinued at P7. Proliferation potentials of AMSCs, CMSCs, and UC-MSCs were assessed over the culture period. Average PDTs of AMSCs, CMSCs, and UC-MSCs at P3 were 68 ± 22.5 hours, 28.2 ± 3.2 hours, and 26.1 ± 2.2 hours, respectively. PDT values of AMSCs, CMSCs, and UC-MSCs increased during the processing of passages (Fig. 1B). AMSCs proliferated slowly when compared to CMSCs (*P* = 0.011) and UC-MSCs (*P* < 0.001). The proliferation activity of CMSCs was comparable to that of UC-MSCs (*P* = 0.110). The percentage of annexin V positive cells was highest in AMSC (26.7%) when compared to those in CMSC (3.9%) and UC-MSC (1.4%) (Fig. 1C). The AMSCs revealed lowest proliferation rate and highest annexin V expression among three kinds of perinatal MSCs. The percentage of SA-β-gal positive cells was also highest in AMSC (87.5%) compared to those in CMSC (10.5%) and UC-MSC (11%) (Fig. 1D).

### Mesodermal differentiation potential of MSCs

We further evaluated the differentiation potential of AMSCs, CMSCs, and UC-MSCs exposed to osteogenic, adipogenic, or chondrogenic induction conditions. After 3 weeks of culturing, MSCs from the three perinatal tissues were able to differentiate into adipocytes, chondrocytes, and osteocytes (Fig. 1E). Adipogenic induction of the three perinatal MSCs was evident by lipid droplets apparent on Oil-red O staining when compared to BM-MSCs. Osteogenic induction showed less accumulation of mineralized matrix in perinatal MSCs by Alizarin Red S staining when compared to BM-MSCs.

### Expression of MSC phenotypical markers

To confirm that isolated and cultured cells from three perinatal tissues corresponded to MSCs^10^, we tested the expression of several MSC phenotypical markers including CD105, CD90, and CD73. As expected, all MSCs regardless of their tissue origin were positive for the expression of mesenchymal markers and negative for hematopoietic lineage markers including CD11b, CD34, CD45, CD79a, and HLA-DR, thus satisfying the criteria for MSC identification (Fig. S1).

### Evaluation of stemness nature using RT-PCR

**Figure 2:**
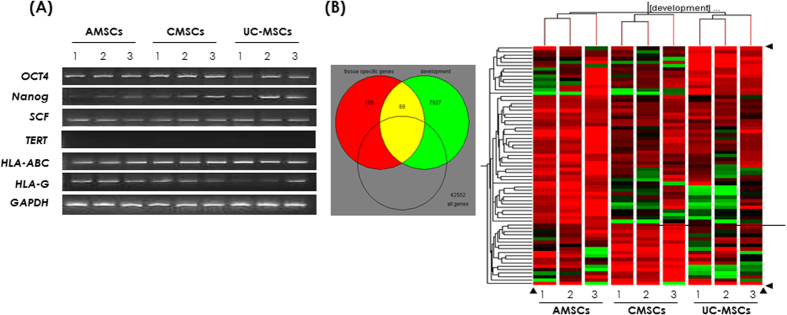
(**A**) Gene expression by RT-PCR of stemness marker, (**B**) venn diagram of 2-fold up-regulated tissue specific and development genes using gene ontology and clustering of genes associated with development.

We observed mRNA expression of genes associated with stemness nature, such as *OCT4, NANOG*, and *SCF*, which are important for the maintenance of stem potential. *HLA-ABC* and *HLA-*G were detected in all samples. *TERT* was not expressed in any samples (Fig. 2A).

### Comparison of gene expression among AMCSs, CMSCs, and UC-MSCs

**Figure 3:**
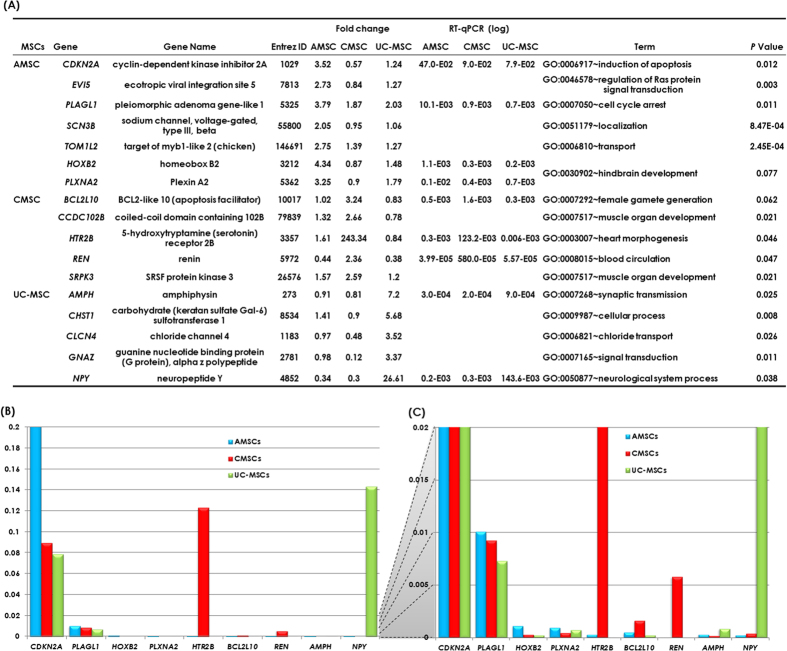
(**A**) Gene ontology analysis for biological processes for mRNAs up-regulated in each mesenchymal stromal cells (MSCs). (**B**) Real-time quantitative PCR showing prominent MSC source-specific gene expression; *CDKN2A* in amnion (AMSCs), *HTR2B* in chorion (CMSCs) and *NPY* in umbilical cord (UC-MSCs).

To examine the gene expression data from microarray analysis, we selected genes with more than 2-fold change in expression on the Fisher’s combined probability test (*P* ≤ 0.01). Of 50,682 genes, 1,190 (2.3%), 509 (1.0%), and 638 (1.3%) were up-regulated in AMSCs, CMSCs and UC-MSCs, respectively. Clustering analysis based on development category demonstrated distinct characteristic gene expression according to the original tissue source, not differing based on individuals (Fig. 2B). Gene ontology (GO) analysis was carried out to characterize the gene expression and was generally classified into several GO terms (Fig. 3A), which are sorted by specifically expressed genes. In particular, genes associated with apoptosis and cell cycle arrest were up-regulated in AMSCs. In CMSCs, genes associated with heart morphogenesis and blood circulation were up-regulated. Genes associated with neurological system processes were up-regulated in UC-MSCs. Real-time quantitative PCR (RT-qPCR) results of the other genes were entirely concordant with the results from gene expression profiles (*CDKN2A, PLAGL1, HOXB2*, and *PLXNA2* in AMSCs, *HTR2B, BCL2L10*, and *REN* in CMSCs, and *AMPH* and *NPY* in UC-MSCs) detected by microarray in their corresponding MSCs (Fig. 3C). Notably, three conspicuous genes were detected which were highly expressed in specific tissue; *CDKN2A* in AMSCs, *HTR2B* in CMSCs, and *NPY* in UC-MSCs (Fig. 3B). *CDKN2A* inhibits CDK4 from participating in cell cycle G1 progression and is associated with growth retardation of MSCs. These results were concordant with those observed by the proliferation and Annexin V expression in AMSC. *HTR2B*, which is present on cardiomyocytes, smooth muscle cells, fibroblasts, valvular interstitial cells, and endothelial cells, is suspected to be involved in cardiac remodeling^11^,^12^. *NPY* promotes differentiation into a nerve cell by activating amyloid-β^13^. In addition, Ingenuity® pathway analysis (IPA) demonstrated similar results to GO analysis. Top functions of up-regulated genes in AMSC, CMSC and UC-MSC were associated with cellular growth and proliferation, cardiovascular and hematological system development, and nervous system development and behavior, respectively (Fig. S2).

### Capability of cardiomyocytes and neural cell differentiation

**Figure 4:**
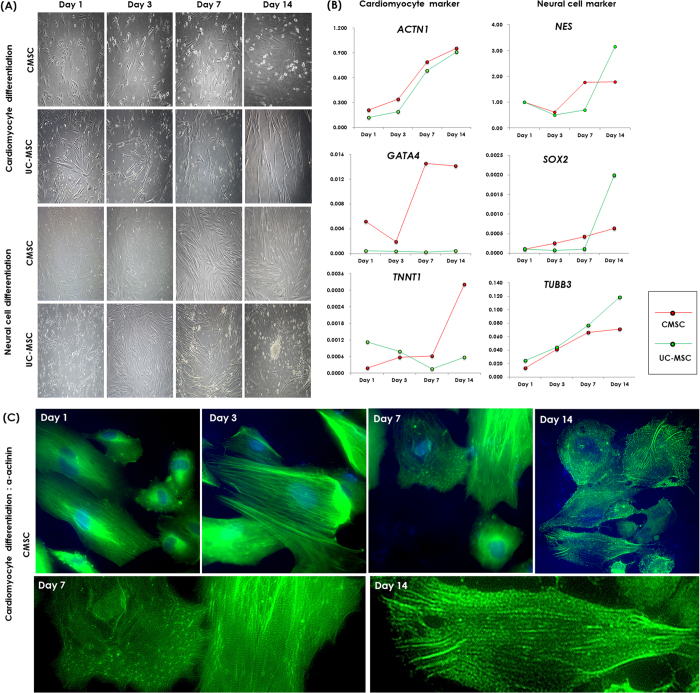
Monitoring the cardiac and neural differentiation of mesenchymal stromal cells (MSCs) from chorion (CMSCs) and umbilical cord (UC-MSCs) by (**A**) morphology (x50), (B) real-time quantitative PCR and (**C**) alpha actinin immunofluorescent staining (upper, x100, lower, x200) at day 1, 3, 7 and 14.

**Figure 5:**
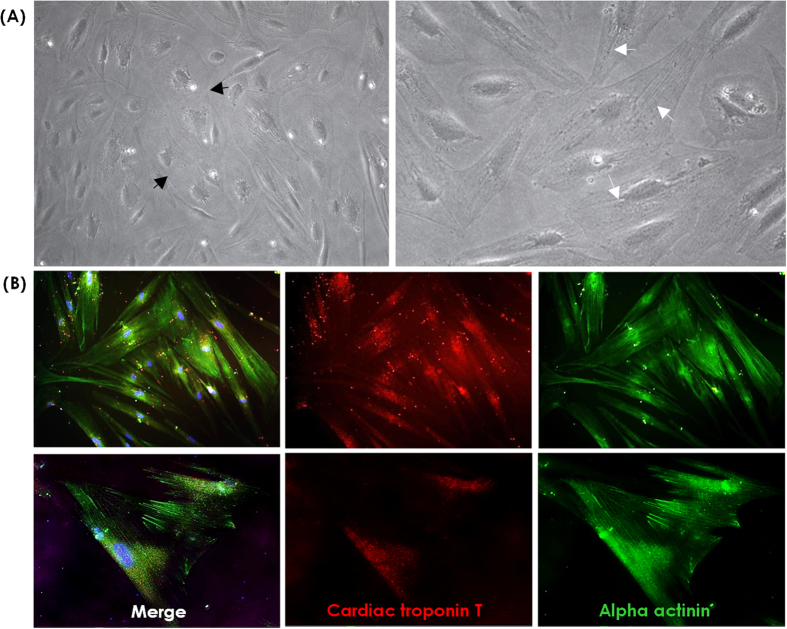
Cardiomyocyte differentiation of chorionic mesenchymal stromal cells. (**A**) The dark arrows show well-defined mature focal adhesions. The white arrows point to stained stress fibers (x100). (**B**) Immunofluorescent staining of chorionic mesenchymal stem cell-derived cardiomyocytes (x200). Merged image (left) of cardiac troponin T (red, middle) and alpha actinin (green, right).

**Figure 6:**
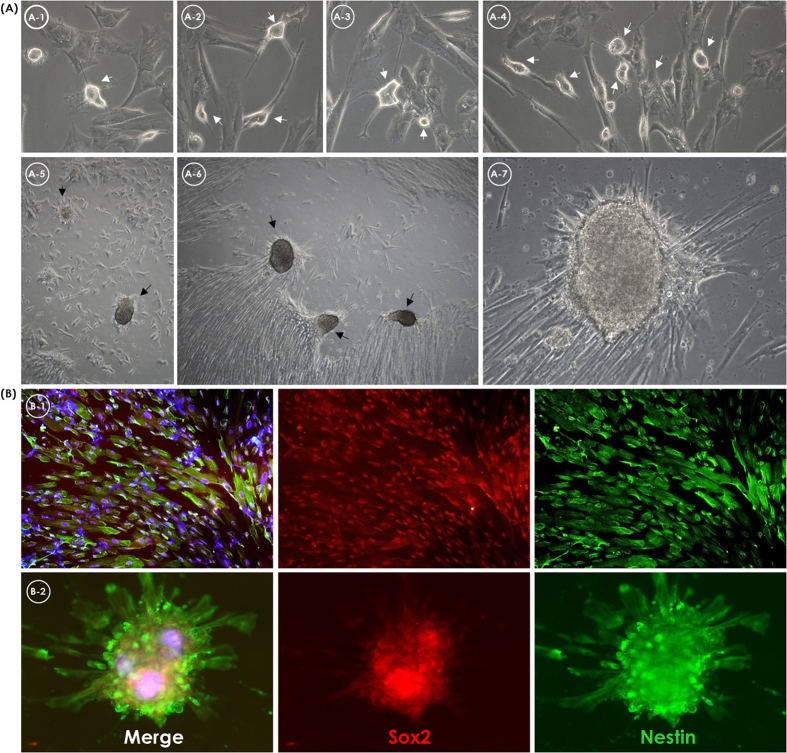
Neural cell differentiation of umbilical cord mesenchymal stromal cells. Neural-like morphology with coarctate bodies and process-like extensions. (**A**) The dark arrows exhibit highly refractive cell bodies with prominent process-like structures (A-1,2,3,4,7; x200, A-5,6; x50). (**B**) Immunofluorescent staining of neural markers of UC-MSC post-differentiated (B-1; x200, B-2; x100). Merged image (left) of sox2 (red, middle) and nestin (green, right).

According to gene expression data, we evaluated the potentials of CMSCs and UC-MSCs to differentiate into cardiomyocytes and neural cells, respectively. The innate potentials detected by gene expression analysis were assigned based on *in vitro* differentiation induction. CMSCs revealed a more pronounced tendency to differentiate into cardiomyocyte-like cells than other perinatal MSCs and BM-MSCs. The morphological characteristics of cardiomyocyte-like cells developed gradually following one week of differentiation induction of CMSCs (Fig. 4A). The cells had enlarged and displayed increased granular content around their nucleus. Two weeks later, the cells were connected as adjoining cells (Fig. 5A). The cardiomyogenic-specific gene expressions including *GATA4, TNNT1* and *ACTN1* were increased during differentiation in CMSCs (Fig. 4B). Immunofluorescent staining of CMSC-derived cardiomyocyte-like cells revealed positive staining of cardiac markers alpha actinin and cardiac troponin T (Fig. 5B). Cardiac troponin T was gradually accumulated as differentiation progressed in CMSCs (Fig. S3) and alpha actinin presented a time-dependent organization of myofibril tend to make a sarcomeric pattern in CMSCs (Fig. 4C). On the other hand, UC-MSCs revealed a more pronounced tendency to differentiate into neural cells than other MSCs. Within the first 48 hours of neural induction, neuron-like morphological changes appeared in the UC-MSCs. Cytoplasm of the cells retracted to form spherical cell bodies, and two or more cytoplasmic processes developed (Fig. S4). With progression of differentiation, the majority of the UC-MSCs exhibited multipolar morphologies with numerous neuronal processes. One week after neural cell differentiation, the cells aggregated and formed neurosphere-like bodies (Figs 4A and 6A). Serial RT-qPCR demonstrated the higher increment of neuron-specific gene expression of *NES, SOX2* and *TUBB3* in differentiated UC-MSCs compared to those in CMSCs (Fig. 4B). Immunofluorescent staining showed positive expression of Nestin and Sox2 in differentiated UC-MSCs (Fig. 6B). Therefore, a specific potency of the CMSCs and UC-MSCs to differentiate in cardiomyocytes and neural cells, respectively, were apparent as the *in vitro* differentiation results were concordant with the gene expression data.

## Discussion

Perinatal tissues carry various types of stromal/stem cells, which are considered excellent candidates for cell therapy. They possess the genetic and behavioral characteristics of both embryonic and adult stem cells^14^, and cell therapeutic trials have already begun using perinatal MSCs to treat various disorders^5^,^15^. However, few studies have investigated the proliferation efficiency and therapeutic efficacy of each MSC type. Understanding the distinct characteristics of each MSC derived from perinatal tissues is important for their optimal therapeutic application.

In this study, AMSCs, CMSCs, and UC-MSCs were isolated successfully and showed characteristics of typical MSCs including fibroblastoid morphology, immunophenotype, and stemness gene expression. AMSCs were heterogeneous at P0 because initial preparation of amnion contained round-shaped amniotic epithelial cells and spindle-shaped AMSCs, which were replaced only by AMSCs after passaging^16^. AMSCs lost their proliferation capacity at a relatively early passage, though their PDT was prolonged dramatically. This phenomenon can be explained by the higher proportion of apoptotic cells in AMSCs. CMSCs and UC-MSCs displayed the highest proliferation capacity at P3, which decreased during repetitive subculturing^6^,^17^. The differences in proliferation capacity are consistent with results from previous studies, demonstrating that AMSCs have limited *ex vivo* expansion potential and the lowest frequency of fibroblast colony forming units^16^,^18^. All cells isolated from these three sources exhibited a typical set of surface proteins, such as CD73, CD90, and CD105, but lacked hematopoietic markers (CD45, HLA-DR, CD79a, CD34 and CD11b). In addition, perinatal MSCs had the potential to differentiate into adipocytes, chondrocytes and osteocytes, while the mesodermal differentiation capacities were inferior to those of BM-MSCs^18^,^19^. We have shown that the pluripotent stem cell markers *OCT4* and *NANOG* are positively expressed in AMSCs, CMSCs, and UC-MSCs^20^. All three perinatal MSCs express *SCF*, which possibly explains its role in maintaining the undifferentiated stage of MSCs by inhibiting the expression of lineage-specific genes^21^,^22^. The *TERT* gene encodes one component of telomerase, which maintains the length of telomeres. The expression of the *TERT* gene is usually undetectable, except for usually being very high in ESCs^14^. Divergent views based on certain aspects of MSCs include how MSCs from perinatal tissues do not live long in culture, while on the other hand not presenting risks of malignant transformation after transplantation. An interesting finding was that these perinatal MSCs, in particular, did highly express *HLA-G*. HLA-G is expressed selectively on the surface of the extravillous trophoblasts and plays an important role in the adaptations of the maternal immune system during pregnancy to prevent the rejection of the semi-allogeneic fetus^23^, and can have inhibitory effects in all immune cells. The expression of *HLA-G* in the perinatal MSCs signifies their embryonic nature and their potential to become resources in regenerative medicine that are superior to other adult MSCs, such as BM-MSCs^24^. The immunomodulatory property of perinatal MSCs as well as the expression of pluripotent stem cell markers and minimal risk of generating various benign or malignant tumors might enhance their desirability as a stem cell source in cell-based therapy.

A comprehensive insight into the functional diversity and the potential therapeutic applicability of each cell types are more important than investigating their characteristics as MSCs. We searched for distinctive gene expression profiles of MSCs using microarrays and RT-qPCR. Hierarchical clustering demonstrated that MSCs from the same tissue clustered together, which represented that MSCs derived from same origins exhibit distinguishing gene expression profiles. Because we compared the data obtained from the same donors, the differences among AMSCs, CMSCs, and UC-MSCs were independent of the interpersonal genetic background. RT-qPCR marked outstanding genes such as *CDKN2A* in AMSCs, *HTR2B* in CMSCs, and *NPY* in UC-MSCs, as well as confirmed the fidelity of the microarray data. AMSCs highly expressed genes related to apoptosis, cellular aging, cell cycle arrest, and antiproliferative properties^25^,^26^. These findings were substantiated by the high proportion of Annexin V and SA-β gal-positive cells and the proliferation reduction in AMSCs. CMSCs display higher expression levels of genes associated with heart morphogenesis, muscle and organ development, and blood circulation^11^,^12^,^27^,^28^,^29^. UC-MSCs characteristically express genes associated with neurological system development and synaptic transmission^13^. UC-MSCs overexpress some transcripts associated with ectodermal lineage or neuronal development, such as *NESTIN, GFAP*, and *SEMA3A*^5^. Therefore, we hypothesized that CMSCs and UC-MSCs could possess a specific potency to differentiate in cardiomyocytes and neural cells, respectively. This speculation was confirmed by GO and IPA analysis using microarray data and RT-qPCR, and *in vitro* differentiation studies. The cardiomyogenic differentiation potential was highest in CMSCs when compared to the potential of UC-MSCs and BM-MSCs. The phenotype of the differentiated cardiomyocyte was confirmed by cardiac specific genes/protein expression. The marked cardiomyogenic differentiation potential of CMSCs may be attributable to their default characteristics as cardiac precursor cells^30^,^31^. These results suggest that CMSCs might be a promising cellular source for cardiac cell therapy and tissue engineering. Our and previous studies have shown that UC-MSCs have a greater potential for differentiation into neural cell lineages and can be regarded as a promising source for the cell therapy of neurological disorders^28^,^32^,^33^. UC-MSCs already have been used in cell transplantation for the treatment of various nervous system diseases in recent years^34^,^35^,^36^, so that our results provided reasonable evidence of applying UC-MSCs to those trials. In addition, the results of investigating innate specific differentiation potency can be helpful to choose more adequate cell sources for better outcome in specific diseases.

This is the first study regarding the differences of MSCs deriving from three different tissues of the human-term placenta through comprehensive gene expression and *in vitro* differentiation analyses. It was demonstrated that perinatal tissue is an excellent source of MSCs, which have typical MSC characteristics such as proliferation potential, immunophenotype, and differentiation into adipocytes, chondrocytes, and osteocytes. Notably, perinatal MSCs can be superior to BM-MSCs due to their *HLA-G* expression and innate differentiation potency. CMSCs and UC-MSCs possess a more pronounced ability to differentiate into cardiomyocytes and neural cells, respectively, and so are more suitable for cardiologic and neurologic disorders, respectively, while their mesodermal differentiation potentials are inferior to those of BM-MSCs. More focused studies to substantiate the specific nature of MSCs derived from different tissues could be more practical for optimizing stem cell based therapy.

## Materials and Methods

### Ethics statement

Human term placentas were obtained after receiving maternal informed consent. This study was approved by the Institutional Review Board of Seoul St. Mary’s hospital (KC09WZZZ0173). Methods were carried out in accordance with the approved guidelines. We received informed consents from all of the participants.

### Isolation and primary culture of MSCs derived from the human amnion, chorion, and umbilical cord

Human term perinatal tissues (n = 8) were donated from healthy donor mothers upon informed consent. The amnion, chorion, and umbilical cord were physically separated from the placenta on the basis of anatomic location. To isolate AMSCs, the amnion was chopped into tiny pieces and treated with 0.25% trypsin-EDTA (Gibco) at 37 °C, followed by 0.2% collagenase IV (Gibco) at 37 °C. CMSCs were isolated using the aforementioned method, with 0.2% collagenase I (Gibco)^37^. Umbilical cord was stripped manually and small tissue obtained was treated with 0.2% collagenase II (Gibco) at 37 °C, followed by 0.25% trypsin-EDTA (Gibco) at 37 °C, to isolate UC-MSCs. BM-MSCs, which were isolated and cultured in our laboratory, served as control. The isolated cells were cultured in α-modified minimum essential medium (α-MEM; Gibco) in T25 flasks (BD Falcon) at 37 °C in a humidified atmosphere containing 5% CO_2_. The medium was refreshed biweekly until the MSCs reached 70% confluency.

### Proliferation potential analysis

When MSCs reached 70% confluency, cells were harvested using 0.25% trypsin-EDTA and counted using a disposal hemocytometer C-Chip (SystemBükerTürk; Incyto, cheonan, Korea). Passaging was performed in a seeding concentration of 200 cells/cm^2^. Proliferation potential was calculated and presented as the population doubling time (PDT) by using an algorithm available online (http://www.doubling-time.com).

### Annexin V/Propidium Iodide for analysis of cellular apoptosis

Cells were harvested from passage 3 (P3) and analyzed for apoptosis using an Annexin V-fluorescein isothiocynate (FITC) apoptosis detection kit (Abcam, Cambridge, UK) according to the manufacturer’s instructions. The cell suspension containing 1 × 10^5^ cells were incubated at room temperature in the dark with FITC-conjugated Annexin V and propidium iodide (PI). The samples were analyzed on a FACSCalibur cytometer (BD Biosciences, San Jose, CA) and the resulting data were processed using CellQuest^TM^ Pro version 6.0 software (BD Biosciences).

### Senescence-associated β-galactosidase (SA-β-gal) activity assay

Cellular senescence can be analyzed by different expression levels of senescence-associated β-galactosidase (SA-β-gal). SA-β-gal staining was performed using SA-β-gal staining kit (Cell Signaling Technology, Boston, MA, USA) according to the manufacturer’s instructions. Briefly, MSCs were seeded at 300 cells/cm^2^ density in 6-well culture dishes and cultured. After 3 days, cells were stained by β-galactosidase staining solution and incubated in a culture dish at 37 °C overnight. Senescent cells were identified as blue-stained cells under inverted microscope at least 20 random fields.

### *In vitro* mesodermal differentiation

AMSCs, CMSCs, and UC-MSCs were harvested at P3 and seeded at a concentration of 1 × 10^5^/well in a 6-well tissue culture test-plate (Nunc; PuDong, Shanghai, China). The medium was changed to a differentiation medium when cells were at 70% confluency. Cells were incubated at 37 °C for 3 weeks, with the medium replaced biweekly.

#### Adipogenesis

Differentiation was induced by culturing cells in adipogenesis differentiation basal medium (Gibco). After 3 weeks, cells were fixed in 4% paraformaldehyde (PFA) and stained using Oil Red-O (Sigma-Aldrich, St. Louis, MO).

#### Chondrogenesis

2.5 × 10^5^cells were centrifuged 150 × g for 5 minutes in a 15 mL conical tube (BD Biosciences), then cultured with chondrogenesis differentiation basal medium (Gibco). After 3 weeks, cells were fixed in 4% PFA and embedded in paraffin. Sections were stained with 1% alcian blue solution (ScienCell, Carlsbad, CA). After rinsing, nuclei were stained with 0.1% nuclear fast red solution (ScienCell).

#### Osteogenesis

Differentiation was induced by culturing cells in osteogenesis differentiation medium. After 3 weeks, cells were fixed in 4% PFA, stained with 2% Alizarin Red Solution (ScienCell).

### Immunophenotyping analysis

Cells harvested from 3rd passage (P3) were used for immunophenotyping analysis. Mouse anti-human monoclonal antibodies: phycoerythrin (PE)-conjugated CD73, CD90, CD105, CD45, CD11b, CD34, CD79a, and FITC-conjugated HLA-DR (BD Biosciences, San Jose, CA) were used. As a control, isotype PE-conjugated IgG1 and FITC-conjugated IgG2a (BD Biosciences) were used. The cell suspension, containing 1 × 10^6^ cells, were incubated with monoclonal antibodies at room temperature in the dark and fixed with BD Cytofix^TM^ (BD Biosciences). The samples were analyzed on FACSCalibur cytometer (BD Biosciences) and the resulting data were processed using CellQuest^TM^ Proversion 6.0 software (BD Biosciences).

### Reverse transcription polymerase chain reaction (RT-PCR) of stemness markers

Total RNA was extracted from P3 AMSCs, CMSCs and UC-MSCs using the RNeasy^®^ Mini Kit (Qiagen, Hilden, Germany), according to the manufacturer’s instructions. cDNA was synthesized using the Transcript or First-strand cDNA synthesis kit (Roche Applied Science, Mannheim, Germany). RT-PCR was performed to evaluate the stemness nature of the MSCs, including octamer-binding protein 4 (*OCT4*), homeobox transcription factor nanog (*NANOG*), stem cell factor (*SCF*), telomerase reverse transcriptase (*TERT*), *HLA-ABC*, and *HLA-G*. Expression of human glyceraldehyde-3-phosphate dehydrogenase (*GAPDH*) was used as an internal control. PCR products bands were observed under ultraviolet light.

### Gene expression by microarray analysis

Microarray analysis was performed to compare the gene expression profile of AMSCs, CMSCs, and UC-MSCs. Universal Human Reference RNA (Agilent, Cedar Creek, TX) was used as a control mRNA. The quality and quantity of extracted RNA and control mRNA were evaluated using the 2100 Bioanalyzer (Agilent) and ND-1000 spectrophotometer (Nanodrop Technologies, Wilmington, DE). These mRNA were not purified. Microarray analysis was performed using SurePrint G3 Hmn GE 8Χ60K V2 Microarray Kit (Agilent) according to the manufacturer’s instructions. Briefly, 200 ng of total RNA was biotinylated using Low Input Quick Amp Labeling Kit (Agilent) and synthesized to double-stranded cRNA. Biotinylated cRNA was purified and amplified according to the manufacturer’s protocols. Subsequently, 600 ng of cRNA hybridized to GeneChip arrays were scanned using a DNA microarray scanner (Agilent) and expression values were quantified using Feature Extraction software 10.7.3.1 (Agilent). The average fluorescence intensity for each spot was calculated and local background was subtracted with the Agilent Feature Extraction software package. All data normalization and selection of up- and down-regulated genes were performed using GeneSpring GX 7.3 (Agilent). Analyses were performed using averages of normalized ratios to particularly strongly-expressed genes according to AMSCs, CMSCs, and UC-MSCs. Strongly expressed genes were selected based on those with averages of normalized ratios of one origin more than two and those with averages of normalized ratios of two other origins less than two. Functional annotation of genes was performed according to the Gene Ontology^TM^ Consortium (http://www.geneontology.org/index.shtml) by GeneSpring GX 7.3. Gene classification was based on searches performed by GeneCards (http://www.genecards.org/) and DAVID (http://david.abcc.ncifcrf.gov/). Gene network analysis was performed for the representation of relationships among biomolecules by QIAGEN’s Ingenuity® Pathway Analysis (QIAGEN Redwood City, www.qiagen.com/ ingenuity). IPA based on development category demonstrated distinct characteristic gene expressions in AMSC, CMSC and UC-MSC.

### Gene expression by real-time quantitative PCR

To confirm gene expression results obtained with by microarray, we selected nine target genes with 2-fold changes of expression in the array experiment data. The selected genes were cyclin-dependent kinase inhibitor 2A (*CDKN2A*) (HS00923894_m1), pleiomorphic adenoma gene-like 1 (*PLAGL1*) (HS00414677_m1), homeobox B2 (*HOXB2*) (Hs00609873_g1), plexin A2 (*PLXNA2*) (Hs00300697_m1, 5-hydroxytryptamine (serotonin) receptor 2B (*HTR2B*) (Hs00168362_m1), bcl-2-like protein 10 (*BCL2L10*) (Hs00368095_m1), renin (*REN*) (Hs00982555_m1), amphiphysin (*AMPH*) (Hs0104720_m1), and neuropeptide Y (*NPY*) (Hs00173470_m1). Eight pairs of AMSCs, CMSCs, and UC-MSCs were used for RT-qPCR analysis. RT-qPCR was performed using the TaqMan^®^ gene expression assay (Applied Biosystems, Foster City, CA) with Master Mix (Applied Biosystems) according to the manufacturer’s instructions. All reactions were performed using the ABI 7500 Real-Time PCR system (Applied Biosystems). Gene expression levels were estimated in triplicate by 2-ΔΔCt method with human GIAPDH (Hs99999905_m1) level as a reference gene.

### *In vitro* differentiation potential to cardiomyocytes and neural cells

AMSCs, CMSCs, and UC-MSCs recovered at P3 were used to evaluate their potential differentiation into cardiomyocytes and neural cells. The medium was changed to differentiation medium at 70% confluency. Cells were incubated at 37 °C for 2 weeks, with replacement of the medium biweekly.

#### Cardiomyocyte Differentiation

Cells (1 × 10^5^ cells/well) were cultured in 35 mm culture dishes (Thermo Scientific, Pittsburgh, PA). Differentiation was induced by culturing cells in a cardiomyocyte differentiation medium (Gibco). The medium was replaced with cardiomyocyte differentiation medium A (Gibco) at 70% confluency. After 2 days, the cells were shifted to cardiomyocyte differentiation medium B (Gibco), and 2 days later to cardiomyocyte maintenance medium (Gibco). After 1-2 weeks, cells were fixed in 4% PFA and stained with anti-alpha actinin antibody and anti-cardiac troponin T antibody (Abcam), and observed in the dark using an Olympus IX 71 fluorescence inverted microscope.

#### Neural cell Differentiation

1 × 10^4^ cells/well were cultured in 96-well tissue culture test-plates (SPL, Pocheon-si, Korea). Differentiation was induced by culturing cells in neural induction medium with supplement kits. After 1-2 weeks, cells were fixed and stained using a human neural stem cell immunocytochemistry kit (Life Technologies, Carlsbad, CA) according to the manufacturer’s instructions. *NESTIN* and *SOX2* expressions were observed using fluorescence inverted microscopy.

#### Tissue-specific Gene expression

To investigate the change of gene expression during differentiation, we serially extracted RNA from MSCs at day 1, 3, 7 and 14. Cardiomyocyte differentiation markers included alpha-actinin (*ACTN1*) (Hs00998100_m1), transcription factor *GATA*-4 (Hs00171403_m1) and Troponin T (*TNNT1*) (Hs00162848_m1), and neural cell differentiation markers included nestin (*NES*) (Hs00707120_s1), neuron-specific class III beta-tubulin (*TUBB3*) (Hs00801390_s1) and *SOX2* (Hs01053049_s1). RT-qPCR was performed using the TaqMan^®^ gene expression assay (Applied Biosystems) according to the manufacturer’s instructions. All reactions were performed using the ABI ViiA^TM^7 Real-Time PCR system (Applied Biosystems). Gene expression levels were estimated in triplicate by 2-ΔΔCt method with human GAPDH (Hs99999905_m1) level as a reference gene.

### Statistical analyses

Proliferation data were analyzed using Wilcoxon Signed Rank Test. Gene expression data by RT-qPCR were analyzed using the one-way ANOVA test and Kruskal-Wallis method. All tests were two-tailed, and *P* values were considered significant when <0.05. All statistical analyses were performed using MedCalc 12.7.2 software (MedCalc, Mariakerke, Belgium).

## Additional Information

**How to cite this article**: Kwon, A. *et al*. Tissue-specific Differentiation Potency of Mesenchymal Stromal Cells from Perinatal Tissues. *Sci. Rep.* **6**, 23544; doi: 10.1038/srep23544 (2016).

## Supplementary Material

Supplementary Information
