# Supplementary material for: Tissue-specific Differentiation Potency of Mesenchymal Stromal Cells from Perinatal Tissues
Source: Sci Rep. 2016 Apr 5;6:23544. doi: 10.1038/srep23544 (PMC4820697; doi:10.1038/srep23544)
Supplement: Supplementary Information [file srep23544-s1.pdf]

Tissue-specific Differentiation Potency of Mesenchymal Stromal Cells from Perinatal Tissues

Ahlm Kwon[1,*], Yonggoo Kim[1,2,*], Myungshin Kim[1,2,*], Jiyeon Kim[1], Hayoung Choi[1], Dong Wook Jekarl[1,2], Seungok Lee[1,2], Jung Min Kim[3], Jong-Chul Shin[4] & In Yang Park[4]

[1]Catholic Genetic Laboratory Center, College of Medicine, Seoul St. Mary's Hospital, The Catholic University of Korea, Seoul, Republic of Korea

[2]Department of Laboratory Medicine, College of Medicine, The Catholic University of Korea, Seoul, Republic of Korea

[3]NAR Center, Inc., Daejeon Oriental Hospital of Daejeon University, Daejeon, Republic of Korea

[4]Department of Obstetrics and Gynecology, College of Medicine, Seoul St. Mary's Hospital, The Catholic University of Korea, Seoul, Republic of Korea

*These authors contributed equally to this work. Correspondence and requests for materials should be addressed to M.K. (email: microkim@catholic.ac.kr)

Correspondence: Myungshin Kim, M.D, PhD

Department of Laboratory Medicine, College of Medicine, Seoul St. Mary's Hospital, The Catholic University of Korea, Banpo-daero 222, Seocho-gu, Seoul 06591, Republic of Korea

Tel: +82-2-2258-1645, Fax: +82-2-2258-1719, E-mail: microkim@catholic.ac.kr

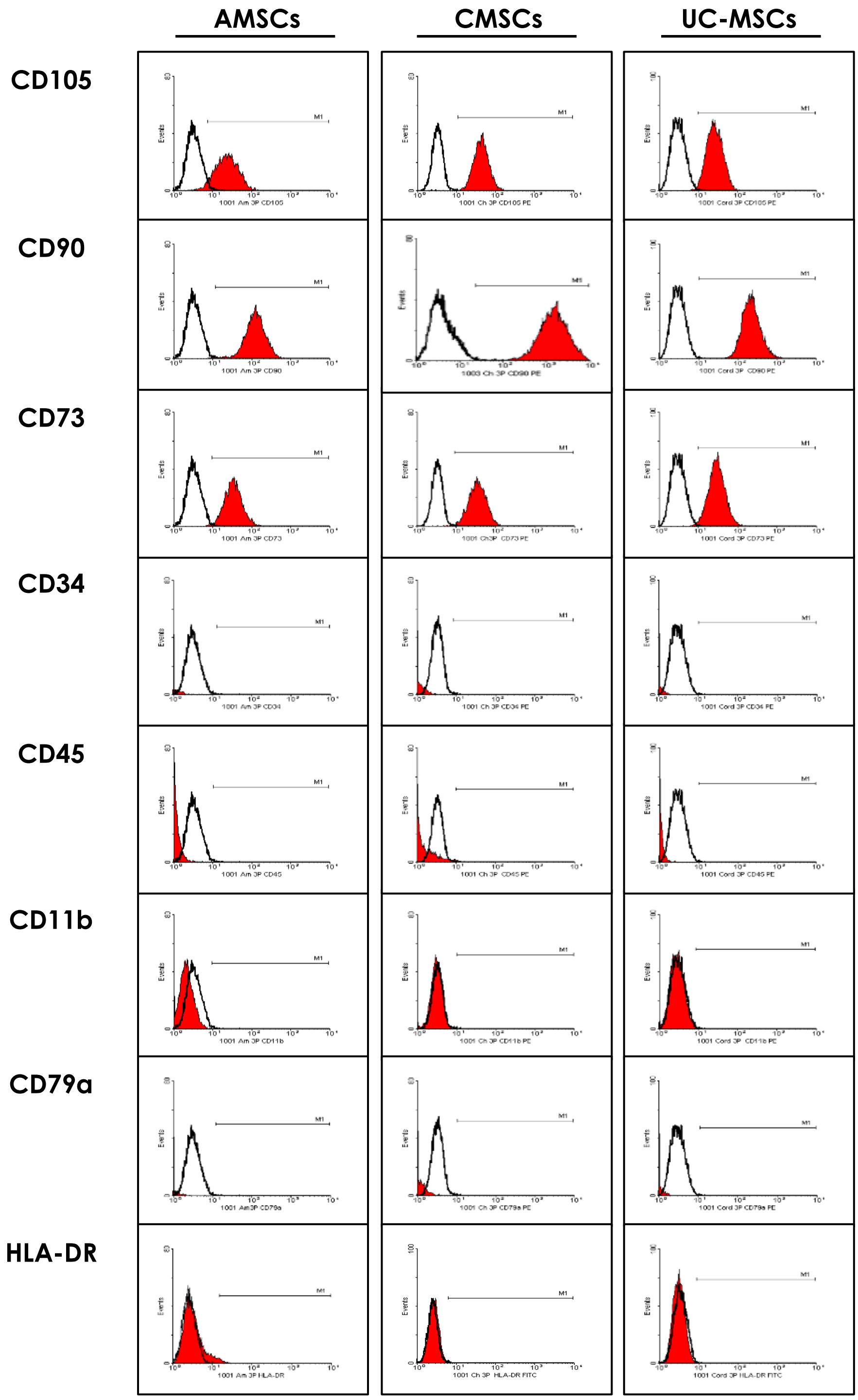

Figure S1. Immunophenotypic expression of amnionic mesenchymal stromal cells (AMSCs), chorionic MSCs (CMSCs) and umbilical cord MSCs (UC-MSCs). Representative flow cytometry histograms showing mesenchymal surface marker expression profile of AMSCs, CMSCs, and UC-MSCs. Red histograms represent antibody staining and black histograms indicate isotype matched IgG controls.

(A)

MSCs	IPA Network Functions	Functions annotation	*P* value	Molecules
AMSCs	Cellular Growth and Proliferation	proliferation of cells	0.00020	*AK4, CCND2, CDKN2A, CLDN1, DCN, GPX3, HAS3, HIPK2, HOXB5, IL18, MEG3, NF1, PCK2, PRKCE, RAPH1, SAA1, SPRY1, STAT4, TXNIP*
		expansion of cells	0.00316	*CCND2, HOXB5, IL18, STAT4*
CMSCs	Cardiovascular System Development and Function	development of cardiovascular system	0.01440	*ALDH1A2,ESAM,HOXD3,HTR2B,MMP3,SIX1,WT1*
		abnormal morphology of cardiovascular system	0.00046	*ALDH1A2,HOXD3,HTR2B,REN,SLC4A1,SLCO2A1,WT1*
		morphogenesis of cardiovascular system	0.01520	*ALDH1A2,HTR2B,SIX1*
		abnormal morphology of heart	0.00142	*ALDH1A2,HTR2B,SLC4A1,SLCO2A1,WT1*
		growth of heart	0.00130	*ALDH1A2,HTR2B,WT1*
		permeability of vasculature	0.00195	*ESAM,MMP3*
		abnormal morphology of artery	0.00308	*HOXD3,REN,WT1*
		pressure of artery	0.00001	*HSD11B1,HTR2B,REN*
		abnormal morphology of heart ventricle	0.00506	*HTR2B,SLC4A1,SLCO2A1*
	Hematological System Development and Function	quantity of macrophages	0.00670	*HSD11B1,MMP3,REN*
		homeostasis of blood	0.00781	*REN,SLC4A1*
		arrest in growth of hematopoietic progenitor cells	0.01230	*WT1*
		function of T lymphocytes	0.01700	*ACKR4,CCRL2,MMP3*
		osmolality of blood	0.02280	*SLC4A1*
UC-MSCs	Nervous System Development and Function	neurotransmission	0.00021	*AMPH,BCHE,KALRN,KCNMA1,NPY,SLC1A3*
		innervation	0.00217	*CDH6,KCNMA1,NTRK3*
		coordination	0.00075	*CPE,KALRN,KCNMA1,NTRK3*
		circadian rhythm	0.02080	*DPYD,KCNMA1*
		chemically-elicited antinociception	0.03560	*GNAZ*
		abnormal morphology of vertebrae	0.02610	*HOXC10,MDFI*
		function of neurons	0.01650	*KALRN,KCNMA1*
		quantity of neurons	0.01190	*KALRN,NPY,NTRK3,SLC1A3*
		memory	0.00156	*KALRN,NPY,NTRK3,SORL1*
		abnormal morphology of neurons	0.00797	*KALRN,NTRK3,SLC1A3,SORL1*
		differentiation of mechanosensory neurons	0.00114	*KCNMA1,NTRK3*
		quantity of neuronal progenitor cells	0.02720	*NPY*
		quantity of sensory neurons	0.00778	*NTRK3,SLC1A3*
	Behavior	learning	0.00020	*AMPH,BCHE,KALRN,NPY,NTRK3,SORL1*
		conditioning	0.00067	*AMPH,KALRN,KCNMA1,NPY*
		behavior	0.00155	*AMPH,BCHE,CPE,KALRN,KCNMA1,NPY,NTRK3,SORL1*
		walking	0.00376	*CEND1,KCNMA1*
		anxiolysis	0.00687	*NPY*
		locomotion	0.03350	*BCHE,CEND1,KCNMA1*

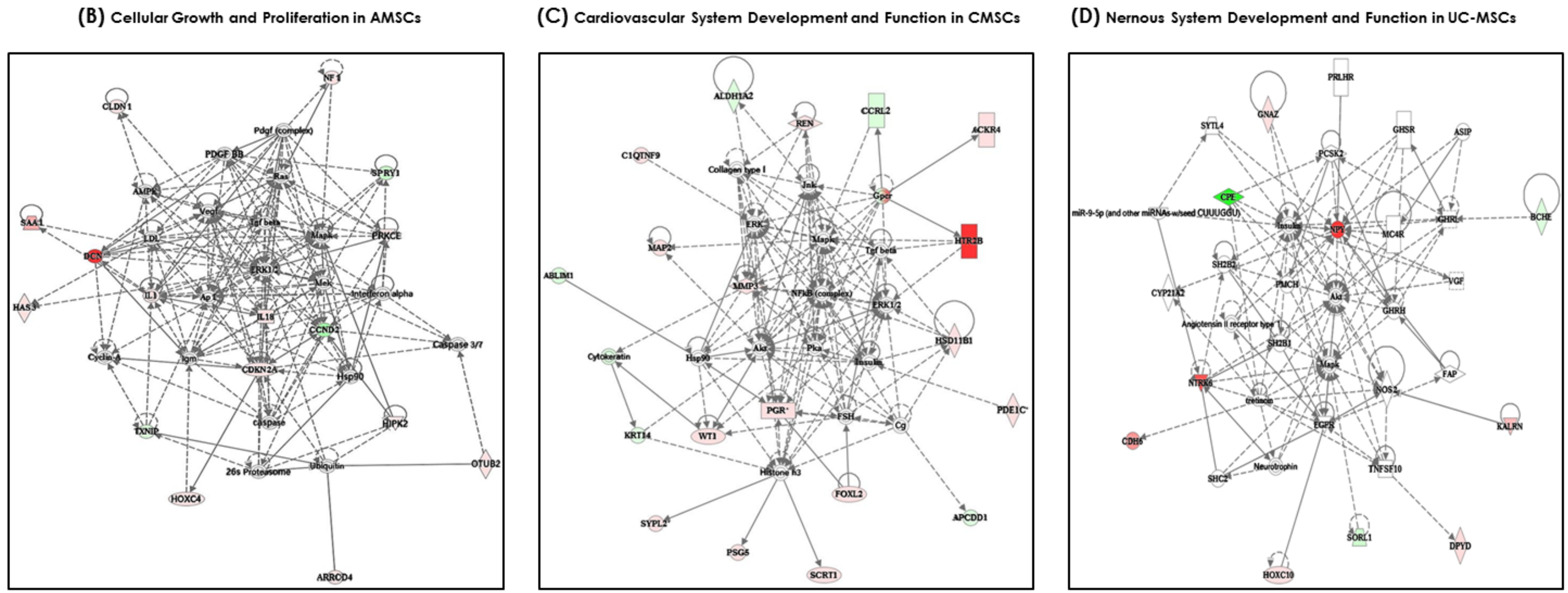

Figure S2. Network analysis. (A) Top networks identified with Ingenuity pathway analysis and representing schematic relationships among key genes changed in (B) amnionic mesenchymal stromals cells (AMSCs), (C) chorionic MSCs (CMSCs) and (D) umbilical cord MSCs (UC-MSCs). The pink or red color nodes in networks indicate a gene that is up regulated and the pale and deep green color nodes do a gene that is down regulate.

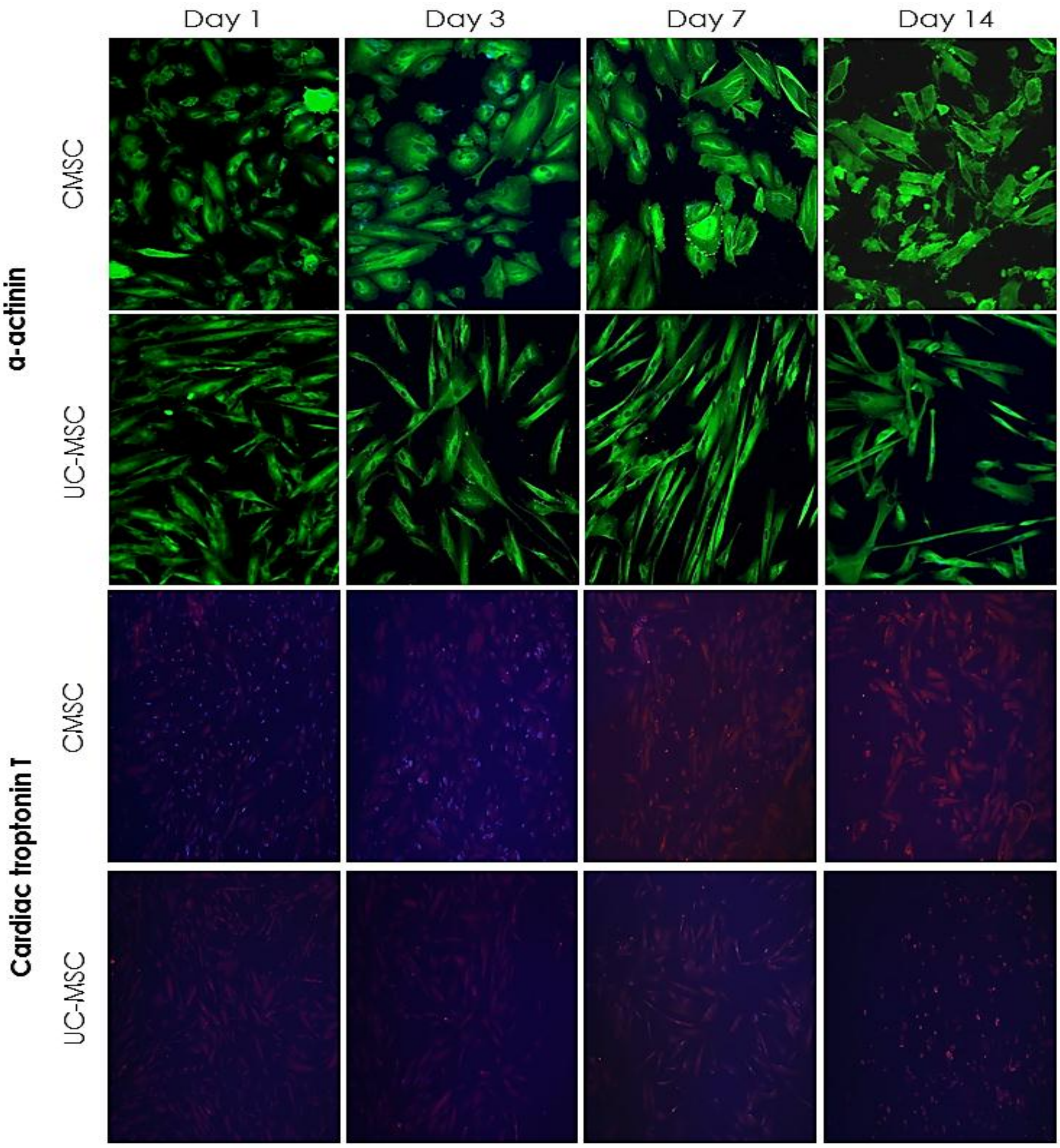

Figure S3. Monitoring the cardiac differentiation of mesenchymal stromal cells (MSCs) from chorion (CMSCs) and umbilical cord (UC-MSCs) by (A) immunofluorescent staining of alpha actinin (green) and cardiac troponin T (red) (x50) at day 1, 3, 7 and 14.

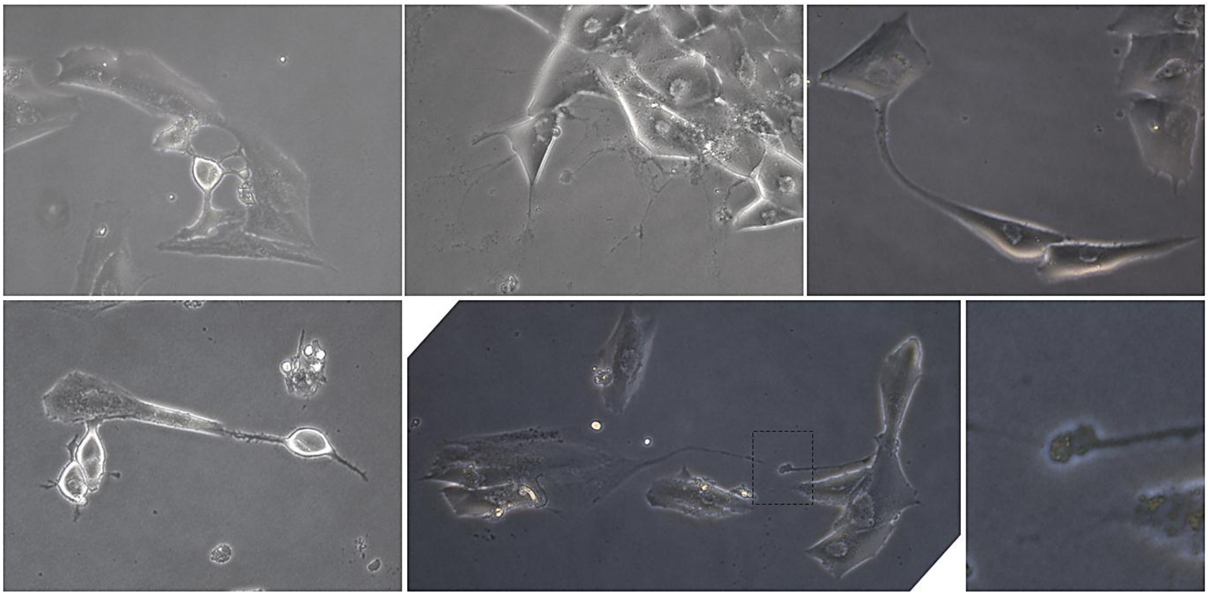

Figure S4. Neuron-like cells with retraction of cell body and long axon-like process after differentiation induction. High magnification phase-contrast view. Note the connected long processes with terminal bulbs (dotted box) (X 100).
